# Doxycycline for Alzheimer’s Disease: Fighting β-Amyloid Oligomers and Neuroinflammation

**DOI:** 10.3389/fphar.2019.00738

**Published:** 2019-07-03

**Authors:** Claudia Balducci, Gianluigi Forloni

**Affiliations:** Istituto di Ricerche Farmacologiche Mario Negri, IRCCS, Milan, Italy

**Keywords:** Alzheimer’s disease, beta-amyloid oligomers, neuroinflammation, tetracycline, memory

## Abstract

Alzheimer’s disease (AD) is the most widespread form of dementia, affecting about 45 million people worldwide. Although the β-amyloid peptide (Aβ) remains the most acknowledged culprit of AD, the multiple failures of Aβ-centric therapies call for alternative therapeutic approaches. Conceivably, the complexity of the AD neuropathological scenario cannot be solved with single-target therapies, so multiple-target approaches are needed. Core targets of AD to date are soluble oligomeric Aβ species and neuroinflammation, in an intimate detrimental dialogue. Aβ oligomers, the most neurotoxic species, appear to induce synaptic and cognitive dysfunction through the activation of glial cells. Anti-inflammatory drugs can prevent the action of Aβ oligomers. Neuroinflammation is a chronic event whose perpetuation leads to the continuous release of pro-inflammatory cytokines, promoting neuronal cell death and gross brain atrophy. Among the possible multi-target therapeutic alternatives, this review highlights the antibiotic tetracyclines, which besides antimicrobial activity also have pleiotropic action against amyloidosis, neuroinflammation, and oxidative stress. A particular focus will be on doxycycline (Doxy), a second-generation tetracycline that crosses the blood–brain barrier more easily and has a safer clinical profile. Doxy emerged as a promising preventive strategy in prion diseases and gave compelling pre-clinical results in mouse models of AD against Aβ oligomers and neuroinflammation. This strongly supports its therapeutic potential and calls for deciphering its exact mechanisms of action so as to maximize its effects in the clinic.

## Introduction

Alzheimer’s disease (AD) is a subtle and so far incurable neurodegenerative disease that makes patients completely unable to run their daily life activities, remember their past and relatives (Selkoe, 2011). It affects about 45 million people worldwide with an enormous socio-economic burden, likely to increase further because of longer life expectancy, and aging as a major risk factor (Garre-Olmo, 2018).

The brains of AD patients present two main lesions: extracellular senile plaques and intracellular neurofibrillary tangles. Senile plaques, rich in aggregates of the β-amyloid peptide (Aβ), act both as a reservoir of the most neurotoxic Aβ soluble species, namely Aβ oligomers (AβOs), and a determinant of neuritic dystrophy and neuronal network interruption (Mucke and Selkoe, 2012). Neurofibrillary tangles are rich in hyperphosphorylated tau protein, which dissociates from microtubules, causing their destabilization. Mitochondria are also compromised (Desler et al., 2018). Neuroinflammation is a chronic neurotoxic event (Heneka et al., 2015), and the vascular system is damaged due to the accumulation of Aβ on the vessel wall (Daulatzai, 2017). On the functional level, synaptic activity is severely impaired and responsible for the onset of cognitive deficits (Mucke and Selkoe, 2012). Progressive neuronal loss culminates in gross brain atrophy (Pini et al., 2016).

The drama of AD lies in the fact that when cognitive deficits arise bringing patients to clinical attention, their brain is already severely compromised and the pathology has progressed for about 10–15 years. This makes the identification of an efficacious therapy a very hard challenge and suggests that the complexity of AD means we must abandon single-target therapies and move on to multi-level approaches. All the single-target “Aβ-centric” clinical trials so far have failed to produce significant benefits (Panza et al., 2019).

The present review will focus on two vital therapeutic AD targets, AβOs and neuroinflammation, that have recently attracted much attention among scientists fighting AD. We highlight the possibility of counteracting AβOs’ detrimental activities and neuroinflammation with doxycycline (Doxy), a second generation-tetracycline antibiotic, which has anti-AβOs, anti-inflammatory activities (Balducci et al., 2018), a good blood–brain barrier (BBB) penetration, and a safe pharmacological profile.

## Aβ Oligomers: The Most Dangerous Synaptic Enemy

The amyloid cascade hypothesis was first put forward by Hardy and Higgins in 1992, stating that: “Deposition of the Aβ peptide, the main component of senile plaques, is the primary event in the pathogenesis of AD” (Hardy and Higgins, 1992). About 10 years later this theory underwent a significant revision, with Aβ plaques overtaken by smaller and soluble AβOs (Hardy and Selkoe, 2002).

AβOs are the first species originating from the amyloidogenic process, by which the Aβ peptide, with its remarked hydrophobicity and overload in AD brains, aggregates, leading to the formation of different-sized polymers including soluble oligomers, protofibrils, and insoluble fibrils.

AβOs are the small, soluble aggregates and the most potent toxic conformers of AD (Haass and Selkoe, 2007), as well as the best correlate of disease severity compared to plaques (Kuo et al., 1996; Lue et al., 1999; McLean et al., 1999). The number of Aβ plaques detectable many years before the onset of clinical symptoms (Perrin et al., 2009) does not correlate with the severity of the cognitive loss in patients (Lue et al., 1999; Naslund et al., 2000) and Aβ deposits are also found in cognitively healthy subjects.

Many experimental data have supported the important role of AβOs in synaptic dysfunction. From transgenic mouse models of AD, it emerged that the onset of synaptic and cognitive dysfunction preceded plaque deposition (Holcomb et al., 1998; Hsia et al., 1999; Mucke et al., 2000; Balducci et al., 2010b). Ultrastructural examination of AD mouse brains revealed the presence of AβOs in the synaptic compartment before plaque deposition (Balducci et al., 2010b). *In vitro* and *in vivo* data also indicated that the application of well-characterized synthetic AβO-enriched solutions, as well as oligomeric species extracted from patient brains or derived from AD mutated cell lines, abolished the formation of new dendritic spines, inhibiting synaptic plasticity and remodeling, thus impairing learning and memory when delivered in the brain of naive mice or rats (Cleary et al., 2005; Lesne et al., 2006; Poling et al., 2008; Shankar et al., 2008; Balducci et al., 2010a; Freir et al., 2011).

Also in humans, biochemical and morphological analyses indicate that AD represents, at least at the more initial stages of the pathology, an attack at the synapses. Indeed, the degree of cognitive decline has been correlated with a decrease of the pre-synaptic marker synaptophysin in the hippocampal area and associated cortices. Notably, a 25% reduction in the expression of synaptophysin was described in the cortex of MCI or very mild AD subjects compared to aged-matched healthy controls (Selkoe, 2002).

In a virtual scenario, AβOs must be visualized as undisturbed dynamic entities, either newly formed or traveling in and out of plaques, perturbing the CNS at many functional levels (Benilova et al., 2012; Forloni et al., 2016).

For many years, synapses have been considered the main AβO target. Initial work supported this, by describing the ability of AβOs to interfere with post-synaptic receptors such as N-methyl D-aspartate receptors (NMDARs) (Balducci et al., 2010b; Yamin, 2009), affecting calcium current, and α-amino-3-hydroxy-5-methyl-4-isoxazolepropionic acid receptors (AMPARs), with the ultimate outcome of inhibition of synaptic plasticity and the induction of memory impairment through the prevention/abolition of new dendritic spine formation where new memories are stored (Chidambaram et al., 2019). Action in the pre-synaptic compartment was also described, through an interaction with the nicotinic acetylcholine receptors α7-nAcChR (Dineley et al., 2001; Puzzo et al., 2008). The cellular prion protein (PrP^C^) has been suggested to mediate AβO effects, although this remains controversial since we and others have confirmed direct binding between PrP^C^ and AβOs, but not a functional contribution (Forloni and Balducci, 2011). Activation of the apoptotic machinery was also described as an AβO-mediated mechanism for synaptic loss (Jo et al., 2011).

However, compelling new theories have emerged in more recent years, bringing to light an intimate mutual interaction between AβOs and glial cells, responsible for synaptic perturbation and loss.

## Neuroinflammation: The Other Special Culprit to Watch Out for

Neuroinflammation has re-emerged as a driving force of neurodegeneration (McManus and Heneka, 2017). Microglia are important in brain tissue homeostasis, secreting neurotrophic factors, and patrolling the microenvironment through the release of cytokines and chemokines that influence astrocytes and neurons, particularly after infection or cell injury. This triggers inflammatory events normally calling a transient immune response followed by tissue repair. Under pathological conditions, resolution (Serhan et al., 2007; Serhan, 2010) can fail, promoting chronic neuroinflammation and neurodegeneration.

Microglia are found in a chronic activated state in AD around senile plaques, and through the continuous release of pro-inflammatory cytokines they drive neuropathology from the very early disease stages (Heneka et al., 2015). Neuroinflammation in the AD brain is chronic presumably because it is never resolved, as indicated by studies showing low levels of specialized pro-resolving mediators (Wang et al., 2015).

A relation between the detrimental action of AβOs and microglial cells is illustrated by the fact that microglia are also crucial in the control of synapse modelling and activity, and consequently of cognitive functions. Resting-state microglia survey neuronal activity by establishing intimate contacts with neurons. They are highly dynamic and plastic cells which continuously extend and retract their processes and contact synapses in an activity- and experience-dependent manner (Morris et al., 2013). However, under pathological conditions, when microglial cell activation is chronic as in AD, synaptic surveillance is lost and cognitive functions are perturbed.

Substantial data have corroborated the existence of a “dangerous liaison” between AβOs and microglial cells, by which they mutually sustain their detrimental effects on synapses. A series of studies comparing the effect of AβOs with that of Aβ fibrils demonstrated that AβOs foster microglial activation to a greater extent and apparently in a conformation-dependent manner (Heurtaux et al., 2010), with the lightest oligomeric species more likely to induce neuroinflammation. Michelucci et al. (2009) reported that AβOs are stronger inducers of the M1 microglial pro-inflammatory phenotype than fibrils. Then, He et al. (2012) described a more pronounced pro-inflammatory action of AβOs after chronic delivery inside the hippocampus of C57BL/6 mice, closely correlated with more severe cognitive deficit, altered neuronal organization, and ultrastructural changes. They also showed that AβOs increased the expression of toll-like receptor-4 (TLR4) and TNFα.

TLR4 belong to a well-known family of pattern recognition receptors initiating the innate immune response and are critically involved in AD (Drouin-Ouellet and Cicchetti, 2012). Using an AβO-induced acute mouse model, we also demonstrated that a single intracerebroventricular injection (ICV) of AβOs in C57BL/6 naive mice induced a transient memory impairment in the novel object recognition test (Balducci et al., 2010a), associated with transient activation of glial cells and an increase in the expression of pro-inflammatory cytokines in the hippocampus within a 2–24 h time window (Balducci et al., 2017), a crucial interval in the elaboration, and consolidation of long-term memory (Sutton and Schuman, 2006). Pre-treatment with anti-inflammatory drugs abolished the AβO-mediated memory impairment. While seeking further insight on the molecular mechanisms linking AβOs and microglial activation toward memory impairment, we also confirmed that TLR4 are vital, since neither memory impairment nor glial activation was observed in TLR4 null mice receiving AβOs ICV (Balducci et al., 2017).

Beside synaptic dysfunction and cognitive deficits, microglia activation also comes on stage to explain synapse loss. In a very elegant paper, Hong et al. (2016) demonstrated that microglia mediates abnormal synapse engulfment through C1q and C3 complement factors. C1q is the initiating protein of the classical complement pathway and, together with C3, localizes on synapses to mediate synaptic pruning through microglial phagocytosis (Presumey et al., 2017). In pathological conditions, such as AD, their expression is increased and localized on post-synaptic proteins, exacerbating synapse loss. The fact that this phenomenon was detectable at very early pre-plaque ages in AD-mutated mice suggested that most likely soluble Aβ species were involved. This was confirmed by specifically injecting AβOs ICV in wild-type mice and demonstrating an increase in C1q synaptic deposition, as well as the C1q-mediated C3 opsonization marking synapses for their elimination (Hong et al., 2016).

## Tetracyclines in the Therapy of AD: Not Only Antibiotics

One of the most difficult challenges in AD is identifying an efficacious therapy to delay the onset, halt its progression, and prevent or reverse cognitive dysfunction. To date most attempts have focused on the Aβ peptide, with scarce or no beneficial effects (Panza et al., 2019). There might be several reasons to explain these multiple failures: wrong treatment timing, inappropriate treatment regimen, and poor or inadequate selection of patients. Although they are all valid possibilities, one of the main problems limiting therapeutic success may lie in the multi-factorial nature of AD, probably requiring multi-target therapies.

We therefore propose the antibiotic tetracyclines as a promising multi-target therapeutic approach, with a special focus on Doxy, a second-generation tetracycline with a safer pharmacological profile and a better passage across the BBB.

Interest in the tetracyclines in AD raised around the early 2000s when it was found, using cell-free approaches, that tetracyclines could inhibit the aggregation of both the synthetic PrP residues 106–126 and 82–146 of human PrP and the Aβ peptide. Facilitation of PrP and Aβ disaggregation as well as the sensitivity of their aggregates to proteases were also described (Tagliavini et al., 2000; Forloni et al., 2001). These anti-amyloidogenic effects were later confirmed for a series of other misfolding proteins responsible for neurodegenerative disorders, including Huntington’s and Parkinson’s disease (reviewed in Stoilova et al., 2013).

Neuroprotective activities of tetracyclines were first demonstrated against PrP *in vitro*, and *in vivo* again using the synthetic PrP residues 82–146 and 106–126 and through infection with the pathological form of the PrP, namely PrP scrapie (PrP^sc^). *In vitro*, tetracycline prevented the PrP 106–126-mediated neurotoxicity and astroglial proliferation (Tagliavini et al., 2000); *in vivo* pretreatment with either tetracycline or Doxy in experimental scrapie reduced infectivity, delayed the onset of pathology, and increased survival when intracerebrally injected in Syrian hamsters (De Luigi et al., 2008). Incubation of 263K PrP^sc^-infected brain homogenate with 1 mM tetracycline or doxycycline resulted in more than 80% reduction in the PK-resistant core of PrP^sc^. It was also reported that these compounds can interact with partially purified PrP^sc^ from patients with the new variant of Creutzfeldt–Jakob disease (CJD) (Forloni et al., 2002).

At the clinical level, Doxy gave positive results in the initial observational studies in CJD patients, which, however, were not confirmed in a double blind against placebo trial in subjects with a diagnosis of definitive or probable sporadic CJD or genetic forms of the disease (Haik et al., 2014). Later it was reported that an asymptomatic CJD patient given Doxy for 4 years survived longer (Assar et al., 2015; Pocchiari and Ladogana, 2015), and Varges and coworkers (2017) showed a longer survival in early-stage CJD patients, suggesting a preventive action of the drug.

In AD, Doxy has been tested in two clinical trials in mild to moderate patients, yielding both positive and negative results (Loeb et al., 2004; Molloy et al., 2013). In the first study there was less decline in cognitive abilities and functional behavior, whereas no benefits were obtained in the second one. The two trials were comparable in terms of patients’ stage of disease at enrolment. Doxy was given orally at the dose of 200 mg/day together with rifampin 300 mg/day in the first study. The same doses were used in the second trial, with the sole difference that Doxy was given at the dose of 100 mg twice a day, rather than one. The main difference lies in the fact that in the former, patients were treated for 3 months, whereas in the latter one, treatment continued for 12 months. As stated by the authors, one possible explanation for the failure in the later study is that Doxy might have some negative properties that become evident when treatment is longer than 3 months. Unfortunately, the therapeutic effects were investigated only at the behavioral and functional levels, with no assessment of Aβ and tau levels in plasma and/or CSF, or of the inflammatory state.

Despite these controversies at the clinical level, preclinical studies indicated the therapeutic potential of Doxy. Initial investigation was done in two simple *in vivo* models. Diomede et al. (2010) tested Doxy in *Caenorhabditis elegans* (*C. elegans*), a simplified invertebrate model of AD where intracellular Aβ deposits caused *C. elegans* paralysis. Doxy protected against this damage by directly interacting with the Aβ aggregates and reducing the load of AβOs. Subsequently, Costa and colleagues (2011) demonstrated that Doxy treatment of Aβ_42_-expressing *Drosophilae melanogaster* did not improve their lifespan but slowed the progression of their locomotor deficits and partially rescued the toxicity of Aβ in the developing eye.

**Figure 1:**
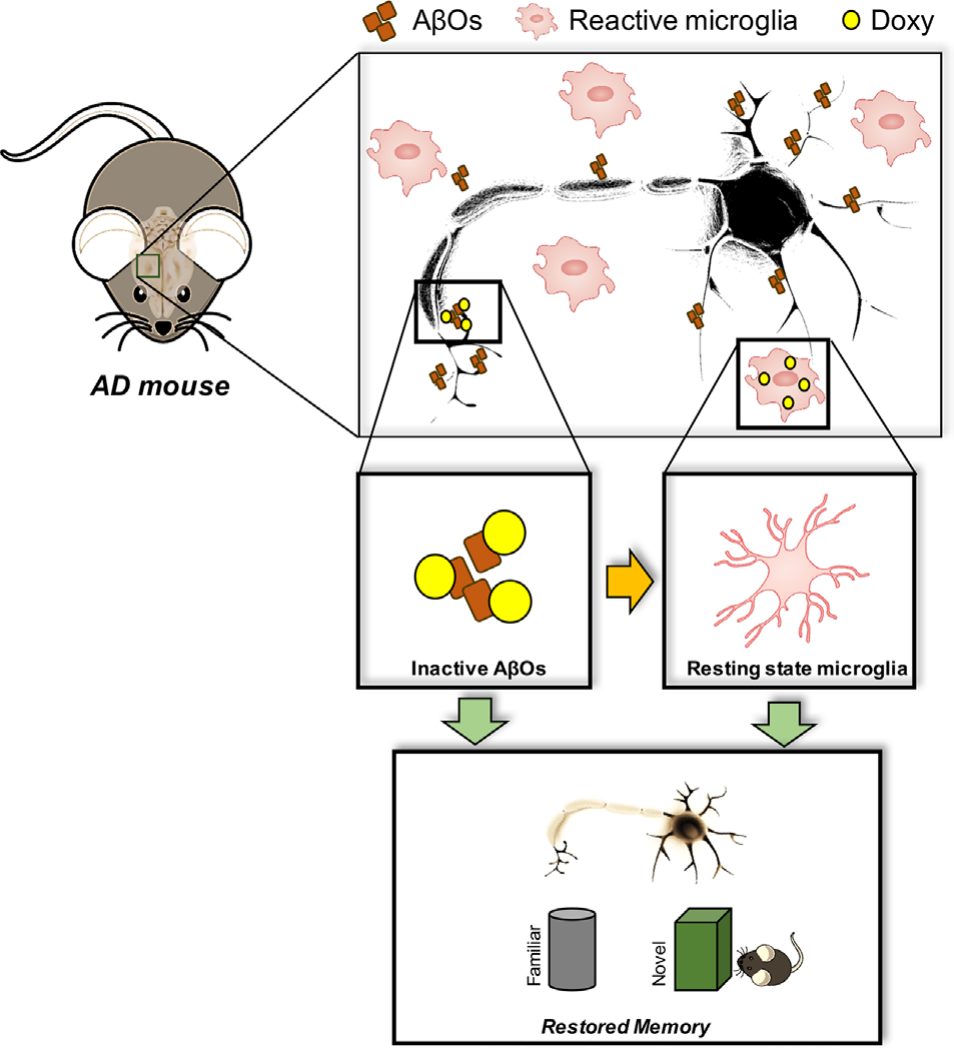
Doxy-mediated effects in AD mouse models. AβOs are the most powerful toxic species in AD brain, which are responsible for the memory impairment. Such detrimental effect is associated with microglial cell activation, a chronic event in AD responsible for both cognitive dysfunction, synaptic loss, and neurodegeneration. Doxy apparently interferes with either the action of AβOs by directly neutralizing their effects at both neuronal and glial level, and/or exerting a direct anti-inflammatory effect. All these actions culminate in a positive outcome at the cognitive level by restoring memory to normal.

We recently found that Doxy had beneficial effects in acute and chronic mouse models of AD (Balducci et al., 2018). Chronic treatment with 10 mg/kg Doxy for 20 days or 2 months, injected intraperitoneally in APP/PS1dE9 transgenic mice, significantly restored memory independently of plaque reduction, but lowered the expression of the 18-mer oligomeric species. Interestingly, an acute treatment also led to memory recovery. On the basis of this evidence, and the lack of changes in plaque number, we assumed that Doxy was restoring memory by interfering with the oligomeric species. This was confirmed in the AβO-induced acute mouse model described above (Balducci et al., 2010a; Balducci and Forloni, 2014), which demonstrated that C57BL/6 naive mice treated ICV specifically with AβOs and pre-treated with Doxy were no longer impaired in their recognition memory. Moreover, because of the close relation between microglial activation and AβO detrimental cognitive effects (He et al., 2012; Balducci et al., 2017), we further show that the memory protection was associated with abolition of AβO-mediated microglial activation. The anti-inflammatory effect of Doxy together with memory recovery was also proved in the APP/PS1dE9 mice chronically treated with Doxy, and in LPS-treated mice, which present an AβO-independent inflammatory context (Balducci et al., 2018). The anti-inflammatory effect of Doxy has been demonstrated in a series of other pathological contexts (reviewed in Stoilova et al., 2013). **Figure 1** depicts all Doxy effects described above in our AD mouse models.

**Figure 2:**
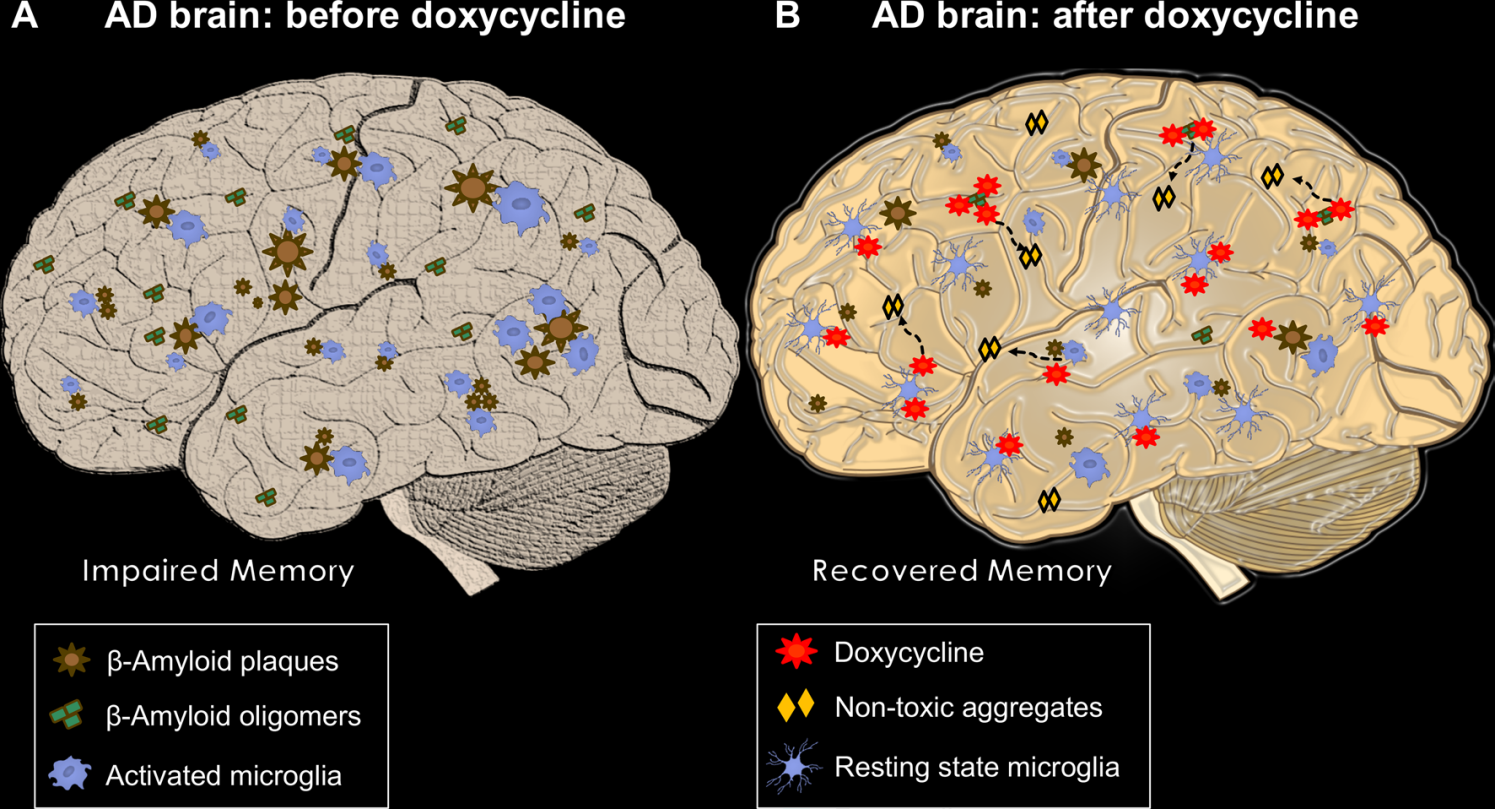
The multiple beneficial actions of Doxy in AD brains. In **(A)**,****an AD brain before treatment with Doxy. Different-sized Aβ plaques are widely deposited with activated microglial cells surrounding them. AβOs are freely circulating entities closer to or far from plaques, which, in concert with neuroinflammation, lead to memory impairment. **(B)** A Doxy-treated AD brain, where the beneficial effects of the drug are summarized. Plaque load can be reduced by long treatment. AβOs interact with Doxy, probably producing non-amyloidogenic and non-toxic structures; microglial cells move closer to a resting state. Both reduction in AβO load and microglial activation may be responsible for the Doxy-mediated memory recovery.

Although we did not find direct AβO-Doxy binding, we assume that—as described for AβOs and tetracycline—an atypical supramolecular interaction might occur, which will result in the formation of colloid structures sequestering and abolishing AβO toxicity *in vitro* (Airoldi et al., 2011). Accordingly, Costa et al. (2011), using transmission electron microscopy, dynamic light scattering, and thioflavin T binding, demonstrated that Doxy leads to the formation of smaller, non-amyloid and non-toxic Aβ aggregates. **Figure 2** summarizes the expected Doxy-mediated changes in the brain of AD patients.

Beside their anti-amyloidogenic and anti-inflammatory effects, tetracyclines also have anti-oxidative and anti-apoptotic activities (Stoilova et al., 2013; Santa-Cecília et al., 2019). Oxidative stress and apoptosis are typical features of AD, whose resolution in the intricate pathological scenario will help to better restore brain physiology.

This evidence and the favorable pharmacological features of Doxy in a translational prospect suggest that this drug holds a considerable therapeutic potential for AD and other neurodegenerative diseases. A recently published comprehensive review describes well the protective effect of Doxy also in Parkinson’s disease and multiple sclerosis (Santa-Cecília et al., 2019).

Despite the beneficial effects of Doxy, clinical trials tell us that not all treatment protocols are effective, or stages of disease adequate for patient enrollment. Patients with too advanced disease are apparently unlikely to respond (Assar et al., 2015; Pocchiari and Ladogana, 2015). This does not necessarily imply that the drug is ineffective, just that it must be used more appropriately. Because of this, Doxy deserves one more chance in AD therapy, more likely with application at a prodromal stage, and a “precision medicine” approach. The latter is strongly recommended, since it will enable us to define the clinical profile (i.e., inflammatory profile) of responders compared to non-responders.

## Author Contributions

CB wrote the manuscript and created figures. GF revised the manuscript.

## Conflict of Interest Statement

The authors declare that the research was conducted in the absence of any commercial or financial relationships that could be construed as a potential conflict of interest.
